# Fasting plus tyrosine kinase inhibitors in cancer

**DOI:** 10.18632/aging.100857

**Published:** 2015-12-06

**Authors:** Irene Caffa, Valter D. Longo, Alessio Nencioni

**Affiliations:** Department of Internal Medicine, University of Genoa, 16132 Genoa, Italy

**Keywords:** cancer, fasting, tyrosine kinase inhibitors, MAPK signaling, molecularly, targeted therapy

Tyrosine kinase inhibitors (TKIs), such as Tarceva (erlotinib), Iressa (gefitinib), Tykerb (lapatinib), and Gleevec (imatinib), are among the most broadly applied cancer therapeutics. By blocking the tyrosine kinase activity of mutated or overexpressed oncogenes, such as Epidermal Growth Factor Receptor (EGFR) and human epidermal growth factor receptor 2 (HER2), they interfere with signaling cascades which cancer cells are frequently “addicted” to, inducing vigorous and prolonged clinical responses in responsive patients [1]. Nevertheless, particularly in solid cancers, patients will sooner or later face relapses due to the emergence of resistant cell clones. Thus, strategies to safely increase the effectiveness of TKIs, but also reduce their toxicity are critically needed.

Studies show that cycles of prolonged fasting (PF, water only for more than two days) or of fasting-mimicking diets (FMDs) enhance the activity of chemo- and radio-therapy in preclinical cancer models [2, 3]. In addition, another advantage of administering chemotherapy during PF is that its overall tolerability appears to be increased [4]. As a result, several clinical trials are currently exploring the effects of PF/FMDs in patients undergoing chemotherapy (NCT01304251, NCT01175837, NCT00936364, NCT01175837, NCT01802346, NCT02126449).

Given this background, it is important to ask whether starvation would also be a useful approach to increase the efficacy of TKIs [5]. Results show that starvation strongly potentiates the antitumor activity of these agents both in vitro and in vivo in mice carrying human tumor xenografts. This goes along with a marked increase in the ability of TKIs to block signaling via the pro-tumorigenic mitogen-activated protein kinase (MAPK) cascade when they are administered under starvation conditions. Gene expression microarrays indicated that starvation and crizotinib (a TKI that is commonly used in advanced non-squamous non-small-cell lung cancer with EML4-ALK translocation) lead to similar changes in gene expression (primarily affecting cell cycle and DNA repair genes), whereas combining the two treatments compounds such effects by activating E2F6 (a dominant negative inhibitor of other E2F family members) and RB1, and by inhibiting the cell cycle-promoting transcription factors E2F1 and E2F4.

Overall, this work indicated that PF and FMDs, recently shown to be effective in reducing IGF-1 levels in both mice and human subjects [6], may not only be effective when coupled to standard chemotherapy or to radiotherapy, but that they may also find applications in patients receiving more modern, molecularly-targeted agents, such as TKIs, making them more effective. That being said, this study also left several questions open and opportunities for investigations. Do PF/FMDs also reduce the likelihood of secondary resistance (or delay its occurrence), thereby extending progression-free survival and overall survival? Can PF/FMDs achieve cases of advanced solid cancers cured with TKIs? Do PF/FMDs also increase the activity of commonly used anti-EGFR and anti-HER2 monoclonal antibodies, such as cetuximab or trastuzumab? Last, but not least, can PF/FMDs also increase the tolerability of TKIs, as much as they do with chemotherapeutics? Indeed, although the toxicity of TKIs is typically less severe that of chemotherapy, it can still be invalidating and lead to dose reductions or treatment discontinuations [1]. Reduced toxicity is anticipated considering the already demonstrated differential regulation of the growth of normal vs. cancer cells by PF/FMDs, which would promote entry of many normal cell types into a non-dividing and protected mode and make them less dependent on tyrosine kinase activity. Thus, if PF/FMDs helped spare healthy tissues from the toxicity of TKIs, the overall effectiveness of these agents could be strongly improved [7]. Answering these questions through preclinical and clinical studies is going to be crucial to provide a clear frame of usefulness for PF/FMDs in oncology.
